# Retraction: GDF8 promotes the cell invasiveness in human trophoblasts by upregulating the expression of follistatin-like 3 through the ALK5-SMAD2/3 signaling pathway

**DOI:** 10.3389/fcell.2026.1974410

**Published:** 2026-09-01

**Authors:** Frontiers Editorial Office

The journal retracts the 28 October 2020 article cited above.

Following publication, concerns were raised on PubPeer regarding the integrity of the images in the published figures. Specifically, Figure 6B and 6D contain GAPDH control western blots, which appear identical to those used demonstrate an a-tubulin control sample in the author’s previously published article:

Hsun-Ming Chang, Jung-Chien Cheng, Elizabeth Taylor, Peter C.K. Leung, Oocyte-derived BMP15 but not GDF9 downregulates connexin43 expression and decreases gap junction intercellular communication activity in immortalized human granulosa cells, Molecular Human Reproduction, Volume 20, Issue 5 May 2014, Pages 373–383, doi: 10.1093/molehr/gau001.

The authors failed to provide a satisfactory explanation during the investigation, which was conducted in accordance with Frontiers’ policies. As a result, the data and conclusions of the article have been deemed unreliable and the article has been retracted.

This retraction was approved by the Chief Executive Editor of Frontiers. The authors do not agree to this retraction.

Frontiers would like to thank Claire Francis for contacting the journal regarding the published article.

